# The feasibility of evaluating outdoor nature-based early childhood education and care provision: a pilot quasi-experimental design

**DOI:** 10.1186/s40814-025-01721-6

**Published:** 2025-11-07

**Authors:** Oliver Traynor, Anne Martin, Nai Rui Chng, Paul McCrorie

**Affiliations:** School of Health and Wellbeing, University of Glasgow, Glasgow, UK

**Keywords:** Feasibility, Pilot, nature, Early education and care, Outdoor play, Evaluation

## Abstract

**Background:**

Systematic reviews have demonstrated the scarcity of well-designed evaluations investigating outdoor nature-based play and learning provision for children in the early childhood education and care (ECEC) sector. This study investigated the feasibility of evaluating outdoor nature-based play and learning provision across urban ECEC settings in Scotland.

**Methods:**

Seventy-seven ECEC settings in Glasgow, Scotland, were contacted to participate in a mixed-methods feasibility and pilot quasi-experimental non-equivalent control study. The evaluation involved ECEC settings with three different models of outdoor nature-based provision. Children aged 3 and 4 years old at participating ECEC settings were eligible. The feasibility of recruitment/retention of ECEC settings and children, propensity score matching in place of participant randomisation, outcome measures, routine monitoring procedures, and study design acceptability were investigated. Outcome measures were completed at baseline and 7 weeks later: anthropometric measures, Strength and Difficulties Questionnaire (SDQ), Preschool Gross Motor Quality Scale, and wrist-worn accelerometer. Feasibility was investigated using descriptive statistics, outcome measure completion rates, and thematic analysis of semi-structured interviews with 15 ECEC educators.

**Results:**

Ten percent (8/77) of ECEC settings expressed a willingness to participate, and six were taken forward for recruitment of children: two traditional ECEC settings, two fully outdoor ECEC settings, and two satellite ECEC (where the outdoor space is not adjacent to the physical premises) settings. Twenty-three percent of children (53/228) provided consent to participate in the study. Data of 46 children were collected at baseline. The retention rate of children at follow-up was 69.8% (37/53). We found few participants were matched on their propensity score. Excluding SDQ assessments and wrist-worn accelerometer, all outcome measures met the green progression category (70% or more completed). Routine monitoring tools, ‘learning journals’, provided a cross-sectional description of how children progress against curriculum outcomes. Practitioners found the pilot study design acceptable in terms of the limited intrusiveness to their daily operations and the level of participation required; however, they suggested that the wrist-worn accelerometers should be reconsidered.

**Conclusion:**

Overall, the study design was accepted by practitioners; however, before an effectiveness evaluation, it is recommended that the recruitment process be improved. The quasi-experimental approach to randomisation should be reviewed considering the outcomes an effectiveness study wishes to investigate (e.g. balance or prosocial behaviour), identification of appropriate covariates, and appropriate sample size. It is recommended that the ‘learning journals’ be used within a case study design monitoring children’s progress against curriculum outcomes. This feasibility study will inform an effectiveness evaluation and support policy making and service delivery in the Scottish ECEC sector.

**Supplementary Information:**

The online version contains supplementary material available at 10.1186/s40814-025-01721-6.

## Key messages regarding feasibility:

1. What uncertainties existed regarding the feasibility?

Outdoor nature-based ECEC is a policy-led programme with several unique uncertainties associated with the feasibility of an effectiveness evaluation: recruitment and retention of ECEC settings and children; the piloting of natural experimental methods to replace participant randomisation; completeness of outcome measures; suitability of routinely collected data to support an evaluation; and the acceptability of trial design methods. These uncertainties were addressed in the present study using pre-defined traffic light progression criteria.

2) What are the key feasibility findings?

Overall, the evaluation of outdoor nature-based ECEC was feasible to implement, and the study design was acceptable to ECEC staff. The recruitment and retention rates met the amber progression criteria, indicating these methods require some minor amendments before being adopted in a full-scale evaluation. Most outcome measures met the green progression criterion; however, the SDQ and wrist-worn accelerometer met the amber and red criterion, respectively. The propensity score matching (PSM) model met the red progression criteria, indicating that the covariates and sample size should be reconsidered before an effectiveness evaluation.

3) What are the implications of the feasibility findings for the design of the main study?

To improve recruitment and retention of ECEC settings and participants, a tailored approach using multiple recruitment strategies specific to the ECEC settings’ context should be adopted. A future effectiveness evaluation of outdoor nature-based ECEC using PSM would benefit from a greater sample size either by recruiting more participants or accessing external population datasets. Other natural experimental methods could be explored, such as difference-in-difference analysis. If the outcomes investigated in this study are still considered important to the research advisory group, then alternative measurement methods should be explored (e.g. thigh-mounted accelerometers). The routine use of ‘learning journals’ could be used in a case-study design to monitor differences in children’s outcomes between ECEC models.

## Background

Globally, it is estimated that 54% of children aged between 3 years and primary school entry age are enrolled in early childhood education and care (ECEC), while in Scotland this figure is considerably higher at 97% [1, 2]. Although the quality of provision can vary considerably, ECEC settings offer the opportunity for a low-cost and equitable population-level health promoting intervention that can have a positive generational impact on children’s wellbeing during a critical time for their development. Providing children with opportunities to spend time outdoors engaging in unstructured play, especially in nature, can support emotional and social resilience, as well as cognitive and physical development [3–5]. Systematic reviews have found inconsistent associations between nature-based ECEC and improved physical activity (PA) levels [4, 6]. Using hip-mounted accelerometers, researchers found a positive but non-significant association where children attending nature-based ECEC engaged in 6 min more moderate-to-vigorous PA (MVPA) compared to children attending traditional ECEC [7], while one study reported a 1-min decrease in MVPA among children engaged in nature-based play [8]. Outdoor nature-based play may also improve motor competence—the degree to which a person is proficient in motor skills like balance and coordination—which is also associated with increased PA [6]. While some studies have reported a positive association between children attending nature-based ECEC and motor competence outcomes like balance [9], others have reported better balance outcomes among children in traditional ECEC [10]. However, both studies used different measurement methods to assess motor competence, making them difficult to compare. Despite these inconsistencies, the affordances of the natural environment, such as woodlands, open fields, vegetation, and hilly terrain, provide rich opportunities for children to explore their surroundings with peers. This type of engagement fosters imaginative and expressive play, which may not only support physical development but also contribute positively to social skills and emotional development [5]. Building on this, a previous Theory of Change developed by the authorship team in collaboration with ECEC stakeholders identified a range of plausible positive influences of outdoor nature-based ECEC on children’s physical, cognitive, social, and emotional development [11].

Nonetheless, most of the research investigating the relationship between nature-based play provision and children’s health outcomes targets older children (greater than 7 years); there is less available evidence for younger children (0 to 7 years) within the early years setting. Available evidence suffers from low methodological quality due to poorly designed evaluations [4–6]. These evaluations are often uncontrolled interventions or cross-sectional studies, with small sample sizes, limited reporting of confounding variables, and minimal reporting of the reasons for participant withdrawal, leading to results with a high risk of bias [5, 6].

Feasibility studies have been conducted in UK ECEC settings before; however, they have focussed on researcher-designed interventions targeting obesity through PA and/or healthy eating [12–14]. Outdoor nature-based play and learning provision is a policy-led programme in Scotland with several unique uncertainties associated with the design of an evaluation. The Medical Research Council guidance on developing and evaluating interventions recommends that study design uncertainties should be investigated in a feasibility study before an effectiveness evaluation [15]. Recently published guidance recommends that such policy-led interventions should be evaluated using natural experimental methods [16]. Example study design methods include interrupted time series design, difference in differences, and propensity score matching [16, 17]. The authors recommend adopting a systematic approach to evaluation planning that engages stakeholders through co-production methods, such as an Evaluability Assessment, and adopting a transparent approach to recording study designs by publishing a study protocol [16].

In Scotland, all 3- and 4-year-old children (and eligible 2-year-olds) are entitled to 1140 h of funded ECEC per year, and it is recommended that children spend as much time outdoors as they do indoors [18]. The ECEC sector provides several formal settings where play and learning take place. They provide different opportunities for exposure to the outdoors with a variety of activities children can engage in while outdoors. These include:

(i) *Fully outdoor* setting, where children spend their time in a forest or park with many natural affordances;
(ii) *Indoor/outdoor setting*, where children can move freely between the indoor and outdoor area of their ECEC setting;
(iii) *Satellite setting*, where the ECEC setting has a nature space (e.g., forest or park), but it is not on their physical premises;
(iv) *Traditional* ECEC setting, often attached to a primary school, where children spend most of their time indoors but with the opportunity to experience the outdoor environment (built and/or natural) as part of structured sessions or play breaks.

The aim of the present study was to determine the feasibility of evaluating outdoor nature-based ECEC provision in Glasgow, Scotland, by addressing key uncertainties and piloting a quasi-experimental design using natural experimental methods.

The study protocol, including the research questions and criteria for progression to an effectiveness evaluation, has been published elsewhere [19]. These publications also include the pre-evaluation stages of programme theory development and an Evaluability Assessment conducted with key stakeholders, which identified several uncertainties associated with evaluation design that will be investigated in the present study [11, 19]. This paper presents data collected during the feasibility and pilot quasi-experimental evaluation of outdoor nature-based ECEC provision and aims to answer the following questions based on our pre-specified progression criteria [19]: (1) How feasible were the recruitment and retention rates of participants? (2) Are natural experimental methods a suitable replacement when participant randomisation is not possible? (3) Were outcome measures sufficiently completed? (4) Could the routine monitoring and evaluation tools in ECEC settings be used in an effectiveness evaluation? (5) Were the study design methods acceptable to ECEC staff members?

## Methods

### Study design

This mixed-methods pilot and feasibility study was conducted to examine the feasibility of recruitment, retention, and data collection procedures, and to pilot a quasi-experimental design using propensity score matching involving six ECEC settings across Glasgow, Scotland: (i) two traditional ECEC settings, (ii) two fully outdoor ECEC settings, and (iii) two satellite ECEC settings. The study took place between April and September 2022. The feasibility and pilot study was reported in line with CONSORT guidelines extended for feasibility trials, Additional file 1 [20]. The study design and choice of child-level outcomes were informed by conversations with the research advisory group, which contained representatives from the local authority, ECEC sector, and third sector.

Ethical approval was provided by the College of Social Sciences, University of Glasgow (no. 400210145) and Glasgow City Council (no. 21.26).

Three changes were made to the original study protocol (see Additional file 2). The eligibility criteria for participating children were expanded to include newly turned 4-year-olds as well as 3-year-olds. Since the study was not powered to detect changes in outcomes, no outcome effect analysis was conducted. Finally, research question five, to what extent is the programme acceptable to ELC managers and practitioners, in the study protocol was published in a separate journal article [21].

### Sample size

This study did not intend to investigate between group differences; therefore, no power calculation was carried out for our sample size. Our target was to recruit two settings of each ECEC model: fully outdoors, satellite, and traditional ECEC settings. To address our feasibility questions, we aimed to recruit 10 children within each participating ECEC setting. To determine the acceptability of the study design, we aimed to recruit the headteacher/manager of each ECEC setting along with two practitioners per setting. The sample size rationale was pragmatic, reflecting the study’s integration within a PhD project subject to logistical and resource constraints, a common approach in early-stage and student-led research [22, 23].

### Participants

Seventy-seven Glasgow-based traditional, satellite, and fully outdoor ECEC settings that operated throughout the year (50 weeks) were invited to participate. Term-time (38 weeks) ECEC settings were not approached since the expected start date of the study was June 2022—the final month before breaking for summer holidays. Within these settings, children and ECEC practitioners were invited to participate in different elements of the feasibility and pilot study. The inclusion and exclusion criteria are outlined in Table 1.

**Table 1:** Inclusion and exclusion criteria for the feasibility and pilot study of outdoor ECEC provision

Participant	Inclusion criteria	Exclusion criteria
ECEC settings	• Local childcare providers (nurseries, family learning centres, and preschools) in the Glasgow City Council area • ECEC settings that operate as traditional, satellite, or fully outdoors	• Childcare settings such as creches, child minders, playgroups, and au pairs/nannies • Not located in Glasgow, Scotland
Children	• Three- or four-year-olds at the time of recruitment or turning three during the study period (May to August 2022) • Must spend at least three sessions per week (morning or afternoon sessions or all day) at the ECEC setting included in the study • Children who have consent from their parents to participate	• Not three or four years old at the time of recruitment or will not be three before the end of the study period (August 2022) • Spend less than three sessions per week at the ECEC under study • Have a serious injury or disability (e.g. wheelchair bound, broken leg, restricted arm movement) that would significantly limit their ability to engage in the study measurement methods • Children whose parent/carer does not provide informed consent to take part
ECEC educators	• Practitioners who supervise the children and support their play while outdoors • Managers/headteachers of participating ECEC settings	• Practitioners who do not spend their working hours outside with the children when the children are outside (e.g. administrative staff)

*ECEC* early childhood education and care

Six ECEC settings were recruited during April/May 2022. Eligible settings were sent an invitation to participate via email. Recruitment of participants took place in settings expressing an interest to take part. Parents/carers (*n* = 53) provided consent for their child to participate by scanning a QR code displayed on recruitment flyers or by completing a paper-based consent form. Scanning the QR code took users to a secure University of Glasgow website. Study data were collected and managed using RedCap (Research Electronic Data Capture) hosted at the University of Glasgow, a secure web-based software platform designed to support data capture for research studies [24, 25]. The website contained the participant information sheet, consent form, demographic survey, and strength and difficulties questionnaire (SDQ). ECEC educators (*n* = 15) provided consent by completing a paper-based consent form.

Upon study completion, participating ECEC settings received a £100 donation and an additional £10 for each educator that participated in the interviews. Parents of children who participated received a £10 shopping voucher after study completion.

### Intervention

ECEC settings representing the different models of outdoor provision (traditional, satellite, and fully outdoors) were recruited to determine the feasibility of evaluating exposure to the natural outdoor environment on children’s health and wellbeing. The ECEC settings operated as normal during the study period; therefore, the intervention was not subject to researcher manipulation. From baseline to follow-up, the study duration was approximately 7 weeks. This timeframe was chosen because the study was not designed to assess effectiveness, and it aligns with similar early years outdoor play interventions such as the 8-week physical activity intervention that demonstrated feasibility and educator acceptability of restructuring outdoor play [26]. This was also a pragmatic choice, as the duration aligned with the summer term, minimising disruption to ECEC settings and families. Measures were taken at baseline (June 2022) and follow-up (August 2022), during which time the ECEC settings were requested to operate as normal. Table 2 provides a summary of the characteristics of each participating ECEC setting demonstrating the differences between the environmental affordances and time spent outdoors between the traditional, satellite, and outdoor ECEC settings.

**Table 2:** Characteristics of participating ECEC settings

ECEC setting	Description	Affordances	Time outdoors
Traditional ECEC 1	Located over two levels in the same building as a primary school. Outdoor area had two spaces accessed via a series of stairs: the ‘playground’ and the ‘garden.’	The playground: selection of manufactured affordances: climbing frame, digger, playhouses, giant tiers, all on artificial surface The garden: smaller space with grass, concrete paths, shed, climbing frame, and mud kitchen	From arrival at 8am, children are given the choice whether they want to be inside or outside. At 10am, children go outside for snacks and lunch is inside at 1130am
Traditional ECEC 2	Standalone building. Outdoor space is located to the rear, accessed through doors	A concrete space closest to the building with bikes, push cars, scooters, rubber tyres, and mud kitchen. At the back, a large grass area with climbing frames, shed, water tables, tree with climbing rope, tight ropes	From 9am, children are given the choice to go outdoors. At 10am, children go inside for snacks and lunch is inside at 11:30am
Satellite ECEC	Satellite space located in a gated area across the street from ECEC premises. Small area under tree canopy. Practitioners usually lead activities	Wooden pallets, hammocks, tyre swing, and mud kitchen	Children had approximately 40 min outside at a time convenient for practitioners
Outdoor ECEC 1	In the morning, children are bused to a local park where they spend all day outdoors. Their ‘base-camp’ is under tree canopy. Children are encouraged to free play	A natural woodland with fallen trees for climbing and open grasslands for running. Practitioners provide some resources such as pots and pans, ‘rainbow sticks’, and ‘story stones’. Practitioners support play by identifying ‘teaching moments’	Children are outdoors in the woods from approx. 10:30am to 2:45 pm
Outdoor ECEC 2	In the morning, children are bused to a local park. Every day, children have the choice to choose a different location in the park	Similar to above, but greater emphasis on children engaging in their own free play with the natural affordances they can find	Children are outdoors in the woods from approx. 10am to 4:30 pm

*ECEC* early childhood education and care

### Feasibility and acceptability outcomes

The assessment of feasibility included examining recruitment, retention, completeness of outcome measures, and the suitability of randomisation methods, using pre-defined traffic light system progression criteria which have been reported and justified in the peer-reviewed study protocol [19], to determine whether modifications are required before progressing to an impact evaluation. A green traffic light category was a strong indication that the method was feasible and can be adopted in an effectiveness evaluation; amber, the method could be feasible with some modifications; and red, the method should not progress without serious modifications and consideration with the research advisory group.

### Recruitment and retention

The feasibility of recruiting and retaining ECEC settings and children was assessed by collecting the following information: number of eligible ECEC settings that were approached to participate in the study; number of ECEC settings that expressed an interest in taking part, declined to take part, and did not respond; number of eligible children who could participate in the study; number of children who provided consent to participate; and number of children who completed baseline and follow-up measures. A green progression criterion for ECE recruitment was defined as at least 30% of contacted settings expressing a willingness to participate, amber 10 to 29%, and red less than 10% of contacted ECE settings.

For participant recruitment, the green progression criterion at the outdoor ECEC settings was defined as at least 50% of eligible children returning a signed consent form, while this was 25% at traditional and satellite settings. The amber progression criterion was defined as at least 50% of ECE settings achieving the green recruitment target; red: amber target not achieved.

For retention within each ECEC model, green was defined as 80% or more participants retained, amber from 50 to 79%, and red less than 50%.

### Propensity score matching

Participant randomisation was not possible in this study because outdoor nature-based provision is a policy-led programme, meaning that it is beyond the control of the researcher, and the study research advisory group did not support potentially limiting children’s exposure to the outdoor environment given that Government guidance suggests ECEC settings should ensure all children are outdoors as much as they are indoors. Consequently, to inform a future effectiveness evaluation aimed at isolating the impact of outdoor nature-based provision on child outcomes, propensity score matching (PSM) was piloted as an as-if randomisation method. The psmatch2 function on Stata version 17 [27] was used for PSM to estimate the effect of a treatment (outdoor ECEC) while accounting for covariates that predicted the treatment [28]. This method matched participants from outdoor ECEC settings (treated) with participants from traditional ECEC settings (control) who had similar propensity scores.

The covariates were collected within the participant demographic survey: weekday time spent outside; weekend time spent outside; gender; age; ethnicity; number of siblings; parent status; mother’s age at birth; Scottish Index of Multiple Deprivation (SIMD) quintile. As an example, children’s balance score was used as the dependent variable.

The findings are presented using the average treatment effect on the treated (ATT). ATT measures the difference in outcomes between those who received the treatment (e.g., outdoor ECEC) and what their outcomes would have been had they not received the treatment. In PSM, ATT was suitable because it compared treated individuals to matched controls with similar propensity scores, effectively controlling for confounding variables and simulating a randomised experiment [29]. This helped isolate the treatment’s effect on those who actually received it, providing a clearer understanding of the treatment’s impact within the observed population.

To improve result validity, matching was based on common support. After matching, psmatch2 facilitates balance checking and treatment effect estimation. Using common support in propensity score matching enhanced the validity of evaluating the impact of outdoor ECEC provision by ensuring the treatment and control groups had overlapping propensity scores [30]. This improved comparability, eliminated unmatched units, and reduced bias, making the groups more similar and approximating an RCT [30]. By focusing on comparable groups, common support increased internal validity and prevented extrapolation bias (i.e. extrapolating to regions where there are no comparable control units) [17]. This approach ensured the estimated effects on child development outcomes were more reliably attributable to the outdoor ECEC programme rather than pre-existing differences between groups.

The progression criteria for PSM in this study were defined as green if 80% or more participants were matched based on propensity scores, amber if 50–79% were matched, and red if less than 50% of successful matches.

### Outcomes

The feasibility of five measurement tools was assessed: weight; height; strengths and difficulties questionnaire (SDQ); Preschool Gross Motor Quality Scale (PGMQS); and physical activity wear time using an Axivity wrist-worn device. Details on the outcome measurement tools have been published previously in the study protocol [19]. Participant recruitment and retention rates were collected. The measures were administered at baseline and follow-up, and completeness was recorded by the research team at both timepoints. Where possible, reasons for withdrawal were also recorded. Demographic data on participating children and ECEC educators were collected at baseline. The study progression criteria were green, indicating that 70% or more measures were returned fully at baseline and follow-up; amber signified a 60% or more completion rate; and red if less than 60% of measures were completed. The first author collected the measures at baseline and follow-up.

#### Anthropometric measures

Children’s weight (to nearest 0.1 kg) and height (to nearest 0.1 cm) were measured twice without shoes, wearing indoor clothing, using a portable stadiometer and calibrated weighing scales. Body mass index (BMI) was derived and *z*-score calculated. Standard operating procedures were followed for both measures at baseline and follow-up. The measures were taken at the beginning of the day at each ECEC setting, before children had their morning snack, and so as not to interfere with the routines of the day.

#### Emotional and behavioural wellbeing

Children’s emotional and behavioural well-being was measured using the validated parent-reported SDQ [31, 32]. The parent-reported SDQ was used to assess the feasibility of parental involvement in an evaluation and to reduce the burden on participating ECEC settings. The SDQ has 25 items divided into five scales with five items each: emotional problems, conduct problems, hyperactivity/inattention, peer relationship problems, and prosocial behaviour. Parents/carers were asked how true statements were about their child using a 3-point scale ranging from 0 (not true) to 2 (certainly true). At baseline, the SDQ was completed on the study website (RedCap) after completion of the demographic survey. A paper version was also available. The SDQ was completed again at follow-up.

#### Balance

Children’s balance was assessed using the Preschool Gross Motor Quality Scale (PGMQS) (33). This is a validated tool for use among 3-year-old children which assesses four components of balance: single-leg standing; tandem standing; walking line forward; and walking line backward [33]. To deliver the test battery, one tape or rope spread across a flat surface 1.5 m long was required. The test battery was piloted with a sample of 3-year-olds before study implementation (19). OT first demonstrated how to perform the task correctly, followed by the children. Each child had one practice trial followed by two scored trials. For each balance task, there were four or five criteria. A score of 0 indicated that the movement was performed incorrectly, while a score of 1 indicated the movement was performed correctly. The highest total balance score was 18. Both scored trials were added to give a total score out of 36. The PGMQS was completed at baseline and follow-up.

#### Physical activity device wear time

The study protocol [19] stated that physical activity, sedentary time, and sleep would be reported in this feasibility study. However, discussions with the research team concluded that, to address the acceptability of wearing the PA device within this population, only wear time was investigated in this study. Children wore a wrist-worn Axivity AX3 triaxial accelerometer on their non-dominant wrist [34]. Acceptability of the wrist-worn accelerometer was first piloted with a sample of 3-year-olds before study implementation [19]. The devices were configured to record data using Open Movement GUI (OMGUI, V1.0.0.43) [35]. Children were asked to wear the accelerometers for 7 days at baseline and follow-up timepoints. The accelerometers were programmed to start recording at the end of the day after anthropometric measures and balance test battery were completed. Parents/carers were asked to ensure their child wore the accelerometer for the requested period, including while sleeping, with a minimum of three consecutive days, removing it only if it caused discomfort or when the child was in water (e.g. swimming or bathing). Parents/carers were given a PA logbook to log when the accelerometer was removed, the reason for doing so, and the time the device was placed back on the child’s wrist. The logbook also contained evaluation questions on the experience of using the accelerometer for the child and parent/carer.

#### Routine monitoring and evaluation tools

The monitoring and evaluation (M&E) tools used within participating ECEC settings were examined to determine whether similar outcomes were recorded across settings. If similarities were present, it could be possible to develop a standardised method of analysing changes in outcomes, reducing participating burden in a future impact evaluation. A sample of M&E tools, called ‘learning journals,’ was collected at each ECEC setting from children who had consent to participate in the study. Examining these tools helps us understand what outcomes are routinely recorded, by whom, and how often. A green progression criterion was achieved if a standardised method of analysing M&E tools was identified that could be used for measuring specific outcomes in an effectiveness evaluation, amber if there was potential for standardisation subject to minor modifications, and red if there was considerable variation between ECE settings regarding the recording of outcomes and modification was considered counterproductive to ECE practice.

#### Study design acceptability

Qualitative semi-structured interviews with ten ECEC practitioners and five headteachers/managers across participating ECEC settings took place after the completion of study measures at the follow-up timepoint. Study design acceptability was assessed by identifying major and minor barriers mentioned by participants regarding the study procedures (e.g. recruitment process, communication between the research team and setting, whether outcome measures were burdensome, etc.). Thirteen interviews took place at the ECEC setting at a time convenient for the participant. Three interviews took place within offices at the University of Glasgow, as this was most convenient for the participants. Audio recordings were transcribed verbatim by a transcription company with data protection agreements with the University of Glasgow. Transcripts were imported to Nvivo version 14 for analysis [36]. Thematic analysis was used to identify themes associated with the suitability and acceptability of the study design methods [37]. A green progression criterion was defined as little to no minor barriers identified by participants regarding the study procedures, amber if three or four modifiable barriers were identified (e.g. modifying recruitment approach), and red if several non-modifiable barriers were identified related to the study procedures.

### Data analysis

Descriptive statistics were reported for the baseline characteristics as frequencies (%) for categorical variables and means (standard deviations) for continuous variables. Where baseline data is missing, this is acknowledged in the findings. Feasibility regarding recruitment, retention, and outcome completion rates was reported as numbers and percentages. The acceptability of the study design was reported using the themes identified through qualitative thematic analysis [37]. Transcripts were first read and re-read for familiarity, and OT designed a coding framework. A deductive coding framework derived from research question five was used focusing on identifying barriers and facilitators to practitioner acceptability of the study design methods.

## Results

### Feasibility

#### ECEC setting recruitment

**Fig. 1:**
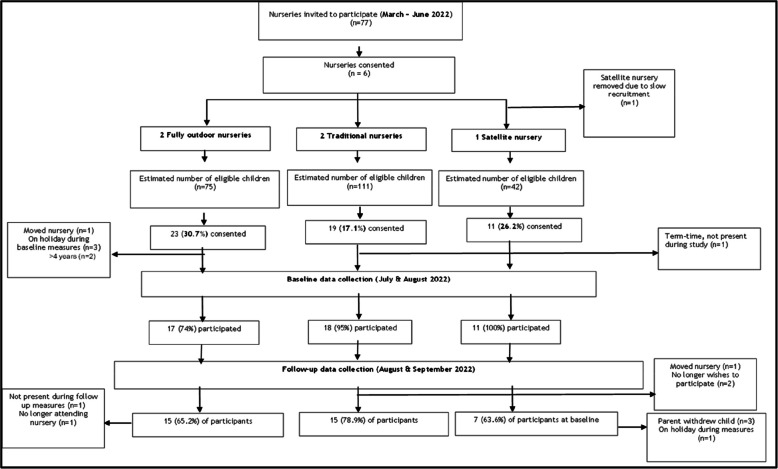
CONSORT Flow diagram. Percentages are of the total number of children who provided consent

Figure 1 summarises participation in the present study, including reasons for withdrawal following the CONSORT guidance. Sixty-nine local authority ECEC settings were contacted and eight private, voluntary, and independent ECEC settings were contacted (*n* = 77). Of the 69 local authority ECEC settings, 45 (65%) were located within the 20% most deprived areas in Scotland as defined by the SIMD [38]. Of the contacted ECEC settings, eight (10.4%) expressed a willingness to participate and seven (9.1%) responded with no interest in participating. This lies within the amber traffic light category for ECEC setting recruitment progression criteria (i.e. at least 10 to 29% of contacted ECEC settings respond with a willingness to participate). Following discussions between the research team and the ECEC settings regarding how they operate their outdoor space, six were taken forward to the child recruitment stage. These six ECEC settings represented the different models of outdoor provision: two fully outdoors; two satellite; and two traditional settings. Reasons for not participating included being understaffed, in the process of moving premises, operating as an indoor/outdoor setting, or not currently using their satellite space. Recovery from the COVID-19 pandemic was often cited as a cumulative factor in these decisions. The study was halted at one satellite ECEC setting due to the lack of participant uptake. Therefore, five ECEC settings participated in the study: two local authority traditional settings; one private outdoor setting; one voluntary sector outdoor setting; and one private satellite setting. Table 3 outlines the characteristics of the participating ECEC settings.

**Table 3:** Characteristics of recruited ECEC settings

ECEC setting	Approximate number of eligible children		SIMD quintile*
	3-year-olds	4-year-olds	
Outdoor ECEC 1	10	19	1
Outdoor ECEC 2	21	25	4
Traditional ECEC 1	11	28	4
Traditional ECEC 2	30	42	2
Satellite ECEC	18	24	1

*SIMD* Scottish Index of Multiple Deprivation

### Participant recruitment

Across all participating ECEC settings, there were estimated to be 228 children eligible to participate in the study. Fifty-three (23.3%) children had consent from their parents/carers to participate in the study. Thirty-seven provided consent via the mobile QR code and 16 used paper-based consent forms. A total of 46 consenting children provided at least one baseline measure. The recruitment and retention of children by ECEC model is demonstrated in Table 4.

**Table 4:** Number of consenting children as a percentage of total eligible children and progression categories

ECEC setting	Approx. No. of eligible children	No. of consenting children *N* (% of eligible)	Traffic light progression category		
			GREEN	AMBER	RED
Outdoor ECECs (*n*=2)	75	23 (30.6)		✓	
Traditional ECECs (*n*=2)	111	19 (17.1)		✓	
Satellite ECEC	42	11 (26.2)	✓		
TOTAL	228	53 (23.2)			

Of the 75 potentially eligible children at the outdoor ECEC settings, 23 parents/carers provided consent for their child to participate. Two children were too old to be eligible for this study, and one child left the ECEC setting before baseline measurements. A further three children were on holiday when baseline measures took place. Therefore, 17 children attending outdoor ECEC settings participated in baseline measures. Across the outdoor ECEC settings, the mean age of participants was 44.8 months (39–54 months, SD = 4.8).

At the traditional ECEC settings, of the 111 potentially eligible children, 19 parents/carers provided consent for their child to participate. One child attended only during term-time; therefore, they were not present during the study period. Eighteen children attending traditional ECEC settings participated in baseline measures. The mean age was 43.4 months (38–50, SD = 3.6).

There were 42 potentially eligible children at the satellite setting; 11 children received consent from their parents/carers to participate in baseline measurements. The mean age was 44.3 months (40–51, SD = 4.5). However, not all participants at the satellite setting returned demographic surveys; therefore, age was not reported for all children participating in the study.

Across participating ECEC settings, a total of 46 children participated in baseline measures between July and August 2022. Table 5 outlines the characteristics of the study sample at baseline. Of the study sample, 48% were female with a mean age of 3.6 years. Most participants were white (76%), had one sibling (46%), and were from a two-parent/carer household (76%). 59% of mothers were aged between 26 and 35 years at the birth of their child. 50% of participants were located within the two lowest quintiles of deprivation (SIMD one and two). However, 15% of participants did not report their postcode; therefore, their SIMD quintiles could not be calculated. Of the diagnosed conditions, two were autism spectrum disorder, one was an eye condition, and one was a dermatological condition. Most participants (76%) spent 15 h or more at their nursery. When not attending nursery, 65% of parents reported that their child spends 3 h or less outside during weekdays. At weekends, 41.3% of parents reported that their child spends 3 h or more outside. The remaining 45.6% spent 3 h or less outside at weekends, while 13% did not provide any data.

**Table 5:** Characteristics of participating children at baseline

Characteristics	All participants	Outdoor ECECs	Traditional ECECs	Satellite ECEC
Participants at baseline	46	17	18	11
Gender (% female)	22 (47.8)	9 (52)	10 (55.6)	3 (27.3)
Age, months (mean, SD)	44.2 (4.2) *(4 missing data)*	44.8 (4.8)	43.4 (3.6) *(1 missing data)*	44.3 (4.5) *(3 missing data)*
Weight, kg (mean, SD)	16.6 (2.1) *(1 missing data)*	16.4 (2.1)	16.5 (2.5) *(1 missing data)*	17.0 (1.5)
Height, cm (mean, SD)	100.9 (4.7) *(1 missing data)*	100.8 (5.0)	99.6 (4.5)	102.8 (4.1)
BMI *z*-score (mean, SD)	0.24 (0.89) *(5 missing data)*	0.22 (0.81)	0.39 (1.07) *(1 missing data)*	− 0.01 (0.68) *(3 missing data)*
Ethnicity (*N*, %)				
White	35 (76.1)	12 (70.6)	16 (88.9)	7 (63.6)
Mixed ethnicity	5 (10.9)	5 (29.4)	0 (0.0)	0 (0.0)
African	2 (4.3)	0 (0.0)	1 (5.6)	1 (9.1)
Other ethnic group	1 (2.2)	0 (0.0)	1 (5.6)	0 (0.0)
Not complete	3 (6.5)	0 (0.0)	0 (0.0)	3 (27.3)
Siblings (*N*, %)				
No siblings	13 (28.3)	5 (29.4)	6 (33.3)	2 (18.2)
1 sibling	21 (45.7)	9 (52.9)	7 (38.9)	5 (45.5)
2 or more	9 (19.6)	3 (17.6)	5 (27.8)	1 (9.1)
Not complete	3 (6.5)	0 (0.0)	0 (0.0)	3 (27.3)
Parental status (*N*, %)				
Single parent/carer	8 (17.4)	3 (17.6)	2 (11.2)	3 (27.3)
2 parent/carer	35 (76.1)	14 (82.4)	16 (88.9)	5 (45.5)
Not complete	3 (2.2)	0 (0.0)	0 (0.0)	3 (27.3)
Mothers age at birth (*N*, %)				
21–25years	6 (13.0)	2 (11.8)	3 (16.7)	1 (9.1)
26–30 years	13 (28.3)	4 (23.5)	6 (33.4)	3 (27.3)
31–35 years	14 (30.4)	5 (29.4)	6 (33.4)	3 (27.3)
36–40 years	9 (19.6)	5 (29.4)	3 (16.7)	1 (9.1)
More than 40 years	1 (2.2)	1 (5.9)	0 (0.0)	0 (0.0)
Not complete	3 (6.5)	0 (0.0)	0 (0.0)	3 (27.3)
SIMD quintile (*N*, %)				
1 (most deprived)	12 (26.1)	5 (29.4)	4 (22.2)	3 (27.3)
2	11 (23.9)	3 (17.6)	4 (22.2)	4 (36.4)
3	8 (17.4)	2 (11.8)	6 (33.3)	–
4	3 (6.5)	1 (5.9)	2 (11.1)	–
5 (least deprived)	5 (10.9)	5 (29.4)	–	–
Not complete	7 (15.2)	1 (5.9)	2 (11.1)	4 (36.4)
Diagnosed condition (*N*, %)				
Yes	4 (8.7)	2 (11.8)	1 (5.6)	1 (9.1)
No	39 (84.8)	15 (88.2)	17(94.4)	7 (63.6)
Not complete	3 (6.5)	0 (0.0)	0 (0.0)	3 (27.3)
Hours at nursery (*N*, %)				
1–5 h	1(2.2)	0 (0.0)	1 (5.6)	0 (0.0)
5–10 h	2 (4.3)	1 (5.9)	0 (0.0)	1 (9.1)
10–15 h	3 (6.5)	3 (17.6)	0 (0.0)	0 (0.0)
15–20 h	13 (28.3)	8 (47.1)	4 (22.2)	1 (9.1)
More than 20 h	22 (47.8)	5 (29.4)	12 (66.7)	6 (54.5)
Not complete	4 (8.7)	0 (0.0)	1 (5.6)	3 (27.3)
Weekday time outside (*N*, %)				
Less than 1 h	10 (21.7)	1 (5.9)	5 (27.8)	4 (36.4)
1–2 h	11 (23.9)	5 (29.4)	4 (22.2)	2 (18.2)
2–3 h	9 (19.6)	3 (17.6)	5 (27.8)	1 (9.1)
3–4 h	6 (13.0)	4 (23.5)	2 (11.1)	0 (0.0)
4–5 h	5 (10.9)	3 (17.6)	1 (5.6)	1 (9.1)
More than 5 h	1 (2.2)	1 (5.9)	0 (0.0)	0 (0.0)
Not complete	4 (8.7)	0 (0.0)	1 (5.6)	3 (27.3)
Weekend time outside (*N*, %)				
Less than 1 h	2 (4.3)	0 (0.0)	1 (5.6)	1 (9.1)
1–2 h	5 (10.9)	2 (11.8)	2 (11.1)	1 (9.1)
2–3 h	14 (30.4)	3 (17.6)	8 (44.4)	3 (27.3)
3–4 h	8 (17.4)	4 (23.5)	3 (16.7)	1 (9.1)
4–5 h	6 (13.0)	3 (17.6)	2 (11.1)	1 (9.1)
More than 5 h	5 (10.9)	3 (17.6)	1 (5.6)	1 (9.1)
Not complete	6 (13.0)	2 (11.8)	1 (5.6)	3 (27.3)

*BMI* body mass index, *SIMD* Scottish Index of Multiple Deprivation

### Participant retention

Follow-up measures took place between five and seven weeks after baseline measures. At follow-up, 37 (80.4%) out of the 46 children at baseline participated in at least one follow-up measure. Table 6 shows the number of children retained at follow-up as a percentage of total children who provided consent (*n *= 53). Two children were not present during follow-up measures, two children were no longer enrolled at the nursery, three children had their consent withdrawn, and two children no longer wished to participate.

**Table 6:** Participant retention and progression criteria as a percentage of consenting participants

ECEC setting	No. of consenting children	No. of children retained at follow-up *N* (% of consenting children)	Traffic light progression category		
			GREEN	AMBER	RED
Outdoor ECECs (*n*=2)	23	15 (65.2)		✓	
Traditional ECECs (*n*=2)	19	15 (78.9)		✓	
Satellite ECEC	11	7 (63.6)		✓	
TOTAL	53	37 (69.8)		✓	

Of the nine participants that were lost at follow-up, four were from the satellite setting (parents of three children withdrew their participation and one was on holiday during follow-up measures), three from traditional settings (one moved nursery and two withdrew their participation), and two from outdoor settings (one was not present during follow-up measures and one no longer attended the ECEC setting); mean age was 41 months (SD = 2.4); 33% were girls. There were no significant differences between the characteristics of participants at follow-up and baseline timepoints.

### Feasibility of propensity score matching

The psmatch2 function was executed on Stata using ‘ECEC model’ (traditional ECEC and outdoor ECEC) as the independent variable. The covariates used were hours enrolled at nursery; weekday time spent outside; weekend time spent outside; gender; age; ethnicity; number of siblings; parent status; mother’s age at birth; and SIMD quintile. To determine the feasibility of PSM, the example dependent variable was children’s balance score using PGMQS. PSMs were based on common support and a nearest neighbour value of one and a caliper distance of 0.2. See Additional file 3 for the output of psmatch2.

**Fig. 2:**
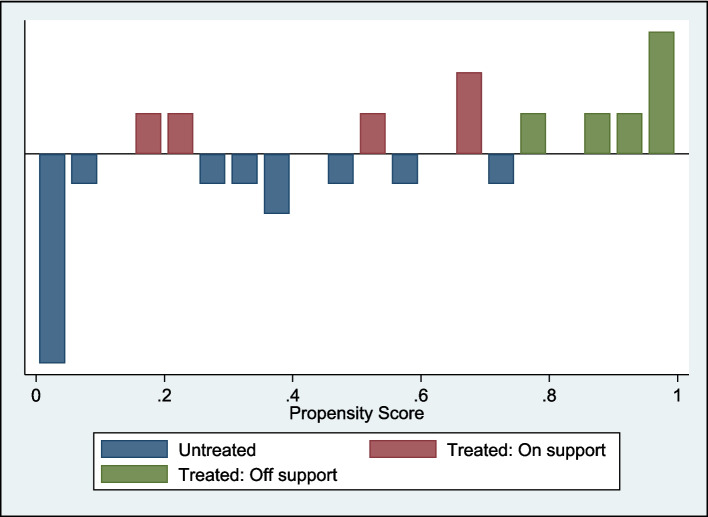
Graphic illustration of propensity score matches with balance as an outcome

The propensity score model revealed substantial differences between outdoor ECEC and traditional ECEC groups. The probit regression estimated treatment assignment based on the covariates outlined above and showed moderate explanatory power (pseudo *R*^2^ = 0.4472); however, the small sample size limited precision. There were 26 observations (11 treated and 15 controls). Approximately 54.5% of outdoor ECE participants (6 out of 11) fell outside the common support region (i.e. treated: off support), indicating a lack of comparable controls (participants from traditional ECE settings). As illustrated in Fig. 2, the region of common support spanned propensity scores of approximately 0.1 to 0.7, with outdoor ECE participants (treated) demonstrating higher mean propensity scores than traditional ECE participants (controls, untreated). The graphic illustration shows a concentration of traditional ECE participants (blue bars) with low propensity scores and outdoor ECE participants (red and green bars) clustered at higher values. The limited overlap restricts the ability to draw causal inferences and reinforces the need for more relevant covariates and a larger sample size.

Therefore, since only five participants from the outdoor ECEC group (treated) were matched with participants from the traditional ECEC group (untreated), this is a red progression category (< 50% of enrolled participants matched).

### Outcome measure completion rates

Excluding SDQ assessments and wrist-worn accelerometers, all assessed measures met the green progression category: demographic survey, weight, height, and PGMQS. Within the traditional and satellite ECEC groups, the SDQ met the amber and red categories, respectively. Across all study groups, the parent-reported SDQ met the amber progression category, suggesting that this measurement tool should be revisited and discussed with the research advisory group before progressing to an impact evaluation. Like baseline, all outcome measures at the follow-up timepoint met the green progression criteria, excluding the parent-reported SDQ and wrist-worn accelerometers, although the study sample was smaller.

#### Accelerometer wear time

At baseline, 42 wrist-worn accelerometers were distributed to participants across the study groups. One (2%) of these devices was lost. Of the 41 devices returned, 15 (37%) malfunctioned; therefore, they did not collect data for their programme period. The malfunctioned accelerometers were replaced with a different accelerometer at the follow-up time point.

At follow-up, 31 accelerometers were distributed to participants across the study groups. Two (6%) were lost. Of the remaining 29, one (3%) malfunctioned; therefore, data were not collected during the programmed period. This meant that of the total devices returned during the study period (*n* = 70), 23% (*n* = 16) malfunctioned.

To address the feasibility question on acceptability of wrist-worn accelerometers in this population, wear time estimates were calculated using the OmGui software (35). Tables 7 and 8 demonstrate the percentage of participants that successfully wore an accelerometer for the requested period at baseline and follow-up. These findings are presented as a percentage of participants who agreed to wear an accelerometer rather than the total number of children present at baseline and follow-up since some participants did not agree to wear the accelerometer. Reasons for not wearing the accelerometer included being off ill, feeling distressed, or not liking the wristband. The mean wear time across all study groups at baseline and follow-up was 5 days. At baseline, all study groups met the red progression category (< 60% outcome completion rate). This was repeated at follow-up, excluding the traditional ECEC group that met a green progression criterion; however, the study sample was smaller compared with baseline; therefore, the findings should be interpreted with caution.

**Table 7:** Percentage of participants that successfully wore accelerometer for study period at baseline

ECEC setting group	number of children that successfully wore accelerometer	Number of children agreed to wear accelerometer	Outcome completion rate (%)	Traffic light progression category
Traditional	8	14	57.1	Red
Outdoor	6	17	35.3	Red
Satellite	1	11	9.1	Red

**Table 8:** Percentage of participants that successfully wore accelerometer for study period at follow up

ECEC setting group	number of children that successfully wore accelerometer	Number of children agreed to wear accelerometer	Outcome completion rate (%)	Traffic light progression category
Traditional	7	10	70	Green
Outdoor	7	14	50	Red
Satellite	3	7	42.9	Red

#### Physical activity logbook responses

At baseline, 61% of logbooks distributed were returned, falling within the amber progression criteria. The most often cited reason for removing the accelerometer was for water-based activities (84%). This was to be expected since the logbook asked parents to remove the wrist-worn device if their child was going to be submerged in water. When asked how their child found the experience, 40% reported a pleasant or positive experience, while 32% did not complete the question. When asked how the parent found the experience of completing the logbook, 44% reported a pleasant or “fine” experience, and 36% did not complete the question. Parents often reported challenges fitting clothes over the watch due to its size and loose strap. One parent suggested a mobile phone app would be better for remembering to take note of when the device was removed from the child’s wrist.

At follow-up, 69% of logbooks distributed were returned, indicating an amber progression category. The most often cited reason for removing the accelerometer was for water-based activities (80%). When asked how their child found the experience, 25% reported a pleasant or positive experience, while 30% did not complete the question. When asked how the parent found the experience of completing the logbook, 40% reported a pleasant or “fine” experience, and 45% did not complete the question. One parent reported how they practice texting each other to remember the times of device removal. Two reported challenges with getting clothes over the device. Finally, one reported confusion as they were not aware their child had to wear the device a second time.

### Feasibility of routine monitoring and evaluation tools

Each ECEC setting had its own version of a ‘learning journal’ to monitor children’s development and record progress against the *curriculum for excellence experience and outcomes* framework [39]. This included literacy, numeracy, health and wellbeing, expressive arts, social studies, science, technologies, and religious and moral education.

The monitoring of outcomes was cross-sectional and descriptive. Recording was completed through direct observation and note-taking by a practitioner, often accompanied by a picture to capture the activity. These were then collated in an MS Word document or on a mobile phone application. Across all ECEC settings, observations occurred sporadically; there was no standardised approach to initiating or implementing observations. The activity was usually recorded in a first-person storytelling approach, cross-referenced with progress on the *experience and outcomes* framework.

Only practitioners at the traditional local authority ECEC settings had a specific group of children assigned to them to ensure that progress on *experiences and outcomes* was recorded. The smaller enrolment at outdoor and satellite settings may have made it easier to share responsibility for monitoring *experiences and outcomes*. However, there was no standardised approach for tracking progress or monitoring outcomes. Additionally, it was not clear whether progress on *experiences and outcomes* for children attending both outdoor and traditional ECEC settings was shared. Most children attending outdoor ECEC settings also spend some of their weekly time at traditional ECEC settings; it is possible that different practitioners at different ECEC settings might record *experiences and outcomes* differently for the same child within different learning journals.

### Acceptability of the study design

Of the interviewed ECEC managers and practitioners, 53% (*n* = 8) were between 18 and 35 years old and 40% (*n* = 6) were between 36 and 62 years old. One participant did not report their age. One participant had less than one year of experience working in the childcare sector. Fifty-three percent (*n* = 8) of participants had two to eleven years of working experience, 20% (*n* = 3) had 12 to 16 years of work experience, and 20% (*n* = 3) had 17 to 26 years of working experience (these were managers). Practitioners working at the traditional ECEC settings had the most experience working in the ECEC sector, while practitioners from the satellite setting had the least years of in-service experience.

The participants found the recruitment and data collection methods of the study design acceptable in terms of the limited intrusiveness to their daily operations and the level of participation required, but they identified challenges in the use of wrist-worn accelerometers. Overall, this indicated a green traffic light progression category. Participants reported that receiving study details in advance allowed them to discuss the study with each other and the children, helping them come to a shared understanding of why the study was taking place and if they wanted to participate:

“[the] fact you emailed everything over to us and gave us time to, sort of, read everything before it, sort of, started and we had time to talk about it… made a big difference.” – Manager, outdoor setting

However, some participants reported that management had not shared the participant information details with them prior to the study commencing. It is possible that this indirectly affected the acceptability of the study design.

Supporting children to wear the accelerometer wristbands was the most referenced challenge of the study.

“I do think they [children] liked the idea of it but then when it was actually on them, for so long…and all the time, like, during the sleep and things like that, I think it was a bit chunky and a bit big. I think…just the wrist band was too big, but I think if it was smaller…in the sense that they were maybe, like, elastic as well so it wasn’t buckled, I think that kind of thing would work.” – Practitioner, traditional setting

The participants suggested that the challenges with wearing the accelerometer were because children were unaccustomed to wearing watches and the long duration required. Similar responses across all ECEC models suggest that the method of accelerometer attachment (i.e., wristband) was not acceptable for children of this age group.

Participants suggested greater parental engagement to improve recruitment and retention, recommending a parents’ evening to introduce the research project before recruitment begins and using routine communication channels to communicate with parents:

“So, like at the end of the day, if you had set up some of your balance beams, or whatever you were doing…and just had the kids interacting… and then the parents are seeing what you're studying, so they're just like, oh he's just watching to see how my kid can walk, that’s not very scary in terms of, like, study. And so I think that…and like, you're there talking with them, I think you probably would have some engagement.” – Practitioner, outdoor setting

“ I did it [communicate with parents] through my Seesaw platform, so again that might be another option, you would… …send it to the children that were going to be involved with you, ‘cause that goes straight to their parents’ phone...” – Manager, traditional setting

## Discussion

This study was designed to address the key uncertainties associated with a full-scale effectiveness evaluation identified in preliminary work conducted by the authors [11, 19]. Guided by the MRC framework for evaluating complex interventions [15], this study’s procedures appear to be mostly feasible for a future impact evaluation. Given that outdoor nature-based ECEC provision is a policy-led programme rolled out across the Scottish early years sector, a future effectiveness evaluation should consider natural experimental approaches to evaluating this population level programme [16]. Findings from this study recommend that careful reconsideration is given to both the selection of covariates and the adequacy of sample size used in the PSM prior to progressing to a full-scale effectiveness evaluation. This is important to ensure that the matching process achieves sufficient balance between groups, minimises bias, and allows for more reliable conclusions regarding the impact of outdoor nature-based ECEC provision on children’s health and wellbeing. Additionally, we recommend that the suitability of wrist-mounted accelerometers for this study population should be reconsidered.

The study was successful in recruiting two ECEC settings per delivery model as per the inclusion criteria outlined in the study protocol [19]. However, the overall response rate of 10.4% lies within the lower end of the amber progression category (10–29% of contacted ECEC settings). This is similar to the response rate of ECEC settings within the Scottish ToyBox feasibility study [14]. Nevertheless, this is lower than the study target of at least 30% response, as demonstrated in previous feasibility studies in British ECEC settings [12, 13].

ECEC-based studies that had a more successful recruitment rate, including the Belgian ToyBox study (27.8% of contacted settings), the NAP SACC UK feasibility study (31.6%), and the Preschoolers in the Playground (PiP) feasibility study (37%), all reported a longer recruitment period and greater initial communication with the local authority and ECEC settings [12, 13, 40]. All the studies had a similar recruitment process to the current study: initial expression of interest followed by emails and phone conversations regarding study details. However, these studies also managed to take advantage of advertising in local authority early years newsletters and presenting at education conferences and events hosted for educators prior to initiating recruitment for their respective interventions [12, 13]. To optimise recruitment, future evaluations of outdoor nature-based ECEC provision should identify similar opportunities and implement a longer recruitment window prior to engaging participants within ECEC settings. Furthermore, leveraging the expertise and networks of the research advisory group could strengthen engagement strategies with both ECEC settings and families, thereby supporting more effective outreach to potential participants.

Regarding participant recruitment, 23.2% of eligible children provided consent to participate in the study, meeting the amber progression category. This is lower than the individual level recruitment rate of the NAP SACC UK feasibility study (35.3%) but higher than the Scottish ToyBox feasibility study (18%) [13, 14]. The overall study retention rate was 80% (20% attrition rate), meeting the green progression category and similar to other studies [13, 14]. However, retention was lower at the satellite setting (64%), meeting the amber progression category. A tailored recruitment approach for participating ECEC settings should be explored for a future impact evaluation.

The opt-in, active, parental consent process demands significant time, energy, and resources from parents, children, schools, and researchers, making it logistically challenging [41]. While active consent is more ethical, it often leads to lower response rates and under-representation of disadvantaged groups, resulting in biased samples and less reliable findings [42]. The ongoing cost of living crisis, COVID-19 recovery, and Brexit put pressure on families, especially in more disadvantaged areas of Glasgow, understandably reducing study participation [43, 44]. To improve recruitment in a future evaluation of outdoor nature-based ECEC provision, a tailored approach is required, involving multiple strategies across several stages from inception to implementation; this should be considered alongside the increased time and cost of research it would bring [45].

We found few participants (*n*=5) were matched on their propensity score, revealing poor covariate overlap between outdoor ECEC and traditional ECEC groups. The PSM in this study was not feasible due to the small sample size available for potential matched controls. PSM requires sufficiently large samples to ensure adequate overlap of covariates between treatment and comparison groups; when samples are limited, bias and variance can increase substantially, reducing the reliability of estimates [29, 46]. Addressing this would require recruiting more participants, which is resource-intensive and often challenging in the early years setting or identifying matched individuals from administrative or population-level datasets. The latter approach, while increasingly used in health and education research, involves governance and access barriers and often constrains the range of outcome data that can be collected [47, 48]. Additionally, it is crucial that there are sufficient data on participant covariates that could influence the outcome of interest to match participants from different exposure groups [49]. In this study, participant’s level of deprivation was determined using the Scottish Index of Multiple Deprivation (SIMD) [38], based on the postcode of primary residence - quintile was used as a covariate within PSM. However, research has shown that area-based deprivation measures like SIMD are often not representative of the populations in these areas [50]. A future effectiveness evaluation of outdoor nature-based ECEC using PSM would benefit from a larger sample size and collecting more specific demographic characteristics such as average household income, level of employment of the primary caregiver, and highest education level of the primary caregiver. These outcomes are known to confound the effect of interventions [51]. A high threshold of around 95% would reduce bias and improve covariate balance. Large representative cohort studies like Growing Up in Scotland could help address this challenge [52]. Additionally, there are other algorithmic approaches that could be adopted (e.g. matching with more than one nearest neighbour) alongside reducing or increasing the caliper distance and considering combining PSM with other statistical techniques such as regression adjustment [49]. It may also be beneficial to explore the use of directed acyclic graphs (DAGs) to identify confounding variables for use in PSM [53]. These approaches should be considered alongside the potential bias that could be introduced to the findings. If caliper distance is increased, this increases the underlying differences between matched participants; therefore, reducing the accuracy of the findings. It is recommended that PSM be carefully reconsidered before progressing to an effectiveness evaluation of outdoor ECEC provision. This includes identification of more relevant covariates and a larger sample size. An alternative natural experimental approach could be piloted, such as difference-in-difference analysis; however, this would require longitudinal data on the participating population groups [16, 54]. Nevertheless, researchers have argued that while sophisticated analytical methods like PSM are useful for demonstrating causality within natural experiments, it is just as important to have a clear theory of change describing the potential mechanistic pathways of the programme under investigation [17]. This helps test explicit causal mechanisms using appropriate evaluation methods.

A green progression category was achieved for the completetion of anthropometric measures and PGMQS, indicating that these measures can proceed to an effectiveness evaluation if they remain of interest to stakeholders and the research advisory group. However, the limitations of the PGMQS in accurately reflecting children’s balance capabilities should be carefully considered. Although children scored low on the PGMQS, this was not reflective of their observed balancing capability during play with their peers. It is the norm to use quantitative measures to assess children’s motor competencies; however, without qualitative data, researchers are unable to understand the context in which children are playing and how this contributes to their balance development [55]. The parent-reported SDQ received an amber progression category, while the wrist-worn accelerometer received a red progression category. It is recommended that the approaches used for these outcome measures be reconsidered before an effectiveness evaluation. For example, more active engagement with parents, opting for teacher-reported SDQs, and trialling a thigh-worn accelerometer along with reducing the required wear time (e.g. removal of the requirement to wear the device during sleep).

The suitability of learning journals for an effectiveness evaluation of outdoor nature-based ECEC provision would depend on the research questions and the outcomes of interest [56]. An important limitation of routinely collected information is that it was not originally collected for the purpose of the present research [57]. Therefore, using such tools requires clearly outlined research questions. If stakeholders and the research advisory group decided that measuring curriculum outcomes was important for a future effectiveness evaluation of outdoor nature-based ECEC provision, learning journals could be used for measuring progress on these outcomes. Monitoring trends in *experiences and outcomes* from a child's enrolment in their ECEC setting through to the start of primary school could facilitate tracking patterns over time and comparing different ECEC settings (e.g. traditional, outdoor, and satellite). For this to be effective, the methods of collecting and analysing observations must be standardised. This would involve the same practitioner recording progress for the same child each time within each setting, and all participating ECEC settings recording progress on the same outcomes at the same time. However, standardising the approach to allow for statistical analysis of children’s development would be counterintuitive to the holistic purpose of monitoring and recording children’s experiences and outcomes described in the *curriculum for excellence* [58].

To improve study understanding and acceptability amongst practitioners, a future study could develop a short video to supplement the participant information sheet [12, 13]. This could be presented during an introductory session between the researchers and staff within the ECEC setting and then shared with staff who were not present [13].

Findings from this study highlight the logistical and resource-intensive challenges of dual recruiting ECEC settings and children/parents. The NAP SACC UK feasibility study also recognised the challenges of this duality and stressed the importance of resources and time [13]. They recommend the adoption of a realistic approach to recruitment for future effectiveness evaluations that recognises the necessity of suitable time and resources, particularly for successful outcome measure completion rates [13].

### Future considerations

This study had several limitations that need to be considered for a future effectiveness evaluation. There were logistical challenges that occurred during the early stages of this study. The decision to only recruit ECEC settings that operated > 50 weeks was a pragmatic decision due to the study commencing towards the beginning of summer, meaning that term-time (38 weeks) ECEC settings would be closed for the summer holidays. This meant that 38-week (school term-time) ECEC settings (*n* = 39) were not contacted to express an interest in the feasibility study since they were close to finishing for the summer holidays (June to August). Also, identifying settings with very low doses of nature exposure was extremely difficult. This was partly because the traditional settings that expressed a willingness to participate had an outdoor play space at their setting that they were proud of and, understandably, wanted to share. However, the satellite setting was the closest to a ‘low dose’ rather than the ‘traditional’ settings. The traditional settings operated more like indoor/outdoor settings with the children having free choice of where to play. This has implications for future evaluation study designs wishing to investigate the differences in outdoor play and learning exposure between ECEC models. The acceptability of the study methods was captured qualitatively. This has the risk of selection bias since it is likely that only practitioners who were positive towards outdoor provision agreed to be interviewed. Finally, future research could explore how specific subgroups, such as children with autism spectrum disorder, might uniquely benefit from nature-based play and learning environments in the Scottish ECEC contexts [59].

## Conclusion

This pilot and feasibility study aimed to address the feasibility of evaluating outdoor nature-based ECEC provision in Scotland by addressing key uncertainties in study design, recruitment, and participant retention. The study successfully demonstrated that the overall design was acceptable to ECEC practitioners and feasible to implement across diverse settings.

Before progressing to a full-scale effectiveness evaluation, a tailored approach to recruitment that involves ECEC settings from the outset is recommended. The use of PSM was not feasible due to the small sample size and insufficient covariate overlap. This highlights the need for a more deliberate approach to identifying covariates that influence both treatment assignment and the outcome of interest, or alternatively, the consideration of a different natural experimental method, such as difference-in-difference analysis. Additionally, outcome measurement tools like wrist-worn accelerometers and parent-reported SDQs require refinement to improve data completeness and acceptability. The use of learning journals shows promise for case study designs to monitor curriculum outcomes. These findings will inform future evaluations and support evidence-based policy development in the Scottish ECEC sector.

## Supplementary Information

Supplementary Material 1.

Supplementary Material 2.

Supplementary Material 3.

## Data Availability

The datasets used and/or analysed during the current study are available from the corresponding author on reasonable request.
