# Supplementary material for: The feasibility of evaluating outdoor nature-based early childhood education and care provision: a pilot quasi-experimental design
Source: Pilot Feasibility Stud. 2025 Nov 7;11:137. doi: 10.1186/s40814-025-01721-6 (PMC12595861; doi:10.1186/s40814-025-01721-6)
Supplement: Supplementary file 2 — Supplementary Material 2. [file 40814_2025_1721_MOESM2_ESM.docx]

### Deviations from published protocol

Three deviations from the original study protocol are outlined below:

1. Participant eligibility criteria were amended to include children who had turned 4-years-old in 2022. Participant recruitment began in June 2022, meaning there were few 3-year-olds enrolled at participating ECEC settings. After discussion with the research team, the eligible age bracket was widened, ensuring the study population was as large as possible while maintaining developmental similarities between children, minimising any impact on the feasibility testing of the study design.
2. As part of the progression criteria for research question three, the study protocol initially stated that outcome effects from baseline to follow-up would be analysed. However, discussions with the study team determined that estimating effects in a study not powered for such analysis would not meaningfully address the feasibility question. Instead, the feasibility assessment of outcome measures focused on participant completion rates.
3. Research question 5 in the study protocol has been published in a standalone peer reviewed article (under review).
