# Supplementary material for: The feasibility of evaluating outdoor nature-based early childhood education and care provision: a pilot quasi-experimental design
Source: Pilot Feasibility Stud. 2025 Nov 7;11:137. doi: 10.1186/s40814-025-01721-6 (PMC12595861; doi:10.1186/s40814-025-01721-6)

Prior to psmatch2 being executed, an estimate of the treatment effect was carried out on STATA version 17 to identify any differences between the study groups (18). A two-sample t-test was carried out to compare the mean value of each outcome between participants in the traditional and outdoor ECEC settings. Since the traditional and outdoor ECEC models had similar sample sizes, these were used as the independent variables. Data from the satellite model was not due to its much smaller sample size. There was no significant difference between the balance scores (p = 0.98) of children in the outdoor and traditional ECEC settings.

Results from psmatch2 with balance (PGMQS) as outcome of interest
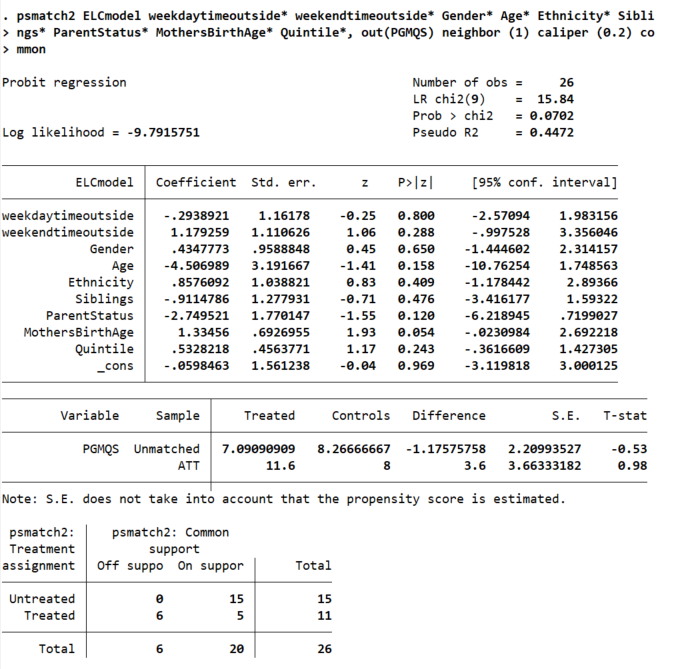

Supplement: Supplementary file 3 — Supplementary Material 3. [file 40814_2025_1721_MOESM3_ESM.docx]
